# Solid Phase Oligo-DNA Extraction from Complex Medium Using an Aminated Graphene/Nitrocellulose Membrane Hybrid

**DOI:** 10.3390/biom14030366

**Published:** 2024-03-19

**Authors:** Georgian Alin Toader, Valentin Titus Grigorean, Mariana Ionita

**Affiliations:** 1 Faculty of Medical Engineering, National University of Science and Technology Politehnica Bucharest, Gheorghe Polizu 1–7, 011061 Bucharest, Romania; georgian.toader3007@upb.ro; 2 Advanced Polymer Materials Group, National University of Science and Technology Politehnica Bucharest, Gheorghe Polizu 1–7, 011061 Bucharest, Romania; 3 Faculty of Medicine, “Carol Davila” University of Medicine and Pharmacy, Boulevard Eroii Sanitari, 050474 Bucharest, Romania; valentin.grigorean@umfcd.ro; 4 Bagdasar Arseni Emergency Clinical Hospital, Berceni Street No. 12, 041915 Bucharest, Romania; 5 eBio-Hub Research Centre, National University of Science and Technology Politehnica Bucharest-Campus, Iuliu Maniu 6, 061344 Bucharest, Romania

**Keywords:** nitrocellulose, fluorescence quenching, DNA extraction, aminated graphene, ions

## Abstract

A hybrid material, consisting of commercially available nitrocellulose (NC) membrane non-covalently modified with amino-polyethylene glycol functionalized reduced graphene oxide (NH_2_-PEG-rGO) nanoparticles, was successfully synthesized for oligonucleotide extraction. Fourier Transform Infrared Spectroscopy (FTIR) confirmed the modification of the NC membrane, revealing characteristic peaks of both compounds, i.e., NC and NH_2_-PEG-rGO. Scanning Electron Microscopy (SEM) exhibited morphological changes in the NC/NH_2_-PEG-rGO hybrid membrane, marked by the introduction of NH_2_-PEG-rGO particles, resulting in a distinctly smothered surface compared to the porous surface of the NC control membrane. Wettability assays revealed hydrophobic behavior for the NC/NH_2_-PEG-rGO hybrid membrane, with a water contact angle exceeding 90°, contrasting with the hydrophilic behavior characterized by a 16.7° contact angle in the NC membrane. The performance of the NC/NH_2_-PEG-rGO hybrid membrane was evaluated for the extraction of ssDNA with fewer than 50 nucleotides from solutions containing various ionic species (MnCl_2_, MgCl_2_, and MnCl_2_/MgCl_2_). The NC/NH_2_-PEG-rGO hybrid membrane exhibited optimal performance when incubated in MgCl_2_, presenting the highest fluorescence emission at 525 relative fluorescence units (r.f.u.). This corresponds to the extraction of approximately 610 pg (≈13%) of the total oligo-DNA, underscoring the efficacy of the pristine material, which extracts 286 pg (≈6%) of oligo-DNA in complex solutions.

## 1. Introduction

In molecular biology and medical research, single-stranded deoxyribonucleic acid (ssDNA) is a fundamental component, playing an essential role in various cellular processes and disease detection [1,2]. Extracting and purifying nucleic acids (DNA and ribonucleic acid (RNA)) with precision and efficiency is of paramount importance as it serves as the foundation for numerous applications in diagnostics, genetic testing, gene expression analysis, and therapeutic interventions. As the demand for accurate and reliable genetic information increases, there is an arising need to develop advanced and reliable extraction methods for ssDNA. Among the various methods used for isolating nucleic acids, such as alkaline lysis, selective hybridization, and ethanol precipitation, the magnetic beads (MB) extraction technique stands out for its non-use of filters and time-efficient procedure [3,4,5]. During the conventional MB technique, an outer magnetic field is utilized to attract and enclose the ssDNA-capturing particles near the tube’s margin, but there is a risk of MB contamination in the eluted sample upon the addition of elution buffer when the magnetic force is turned off [4,6,7].

To address this, a solid-phase extraction method was introduced that is based on nitrocellulose (NC) membranes and offers an alternative method for enhancing the surface adsorption of ssDNA. NC membranes are known for their flexibility, high binding capacity of up to 80 µg/cm^2^, and stable physical and chemical properties. Upon application, ssDNA adheres to the NC membrane through a combination of van der Waals forces, electrostatic interactions, and hydrogen bonding, and can also be covalently linked via chemical cross-linkers or selectively hybridized through sequence-complementary probes. Further developments of the NC membrane extraction method include the modification of NC membranes with graphene-based materials, renowned for their ability to immobilize low concentrations of nucleic acids [8,9,10,11,12].

In our prior study, the combination of NC membranes modified with graphene oxide (GO) nanoparticles was utilized as a solid phase for oligonucleotide extraction. This approach demonstrated favorable outcomes after a 60-min incubation in αMEM, yielding a fluorescent signal equivalent to 7% (330–370 pg) of the total ssDNA, prompting the investigation to extend to other graphene variants [13]. Among the well-known graphene derivatives like pristine graphene, GO, reduced graphene oxide (rGO), graphene nanoplatelets, and graphane, there is another derivative called amino-polyethylene glycol functionalized reduced graphene oxide (NH_2_-PEG-rGO) with great promise in biomedical applications [14,15,16,17,18]. NH_2_-PEG-rGO serves as support material for DNA immobilization in various approaches, such as field effect transistors (FET) [19,20], electrochemical [21,22], and fluorescent-based biosensors [23,24,25]. Among these options, fluorescent biosensors have captured the attention of researchers due to their distinctive characteristics, which encompass selectivity, sensitivity, simplicity, cost-effectiveness, and rapid response [26,27,28]. In a previous study, our group utilized NH_2_-PEG-rGO as a quencher due to its capability to function as an electron acceptor, which suppresses the fluorescence of the marked ssDNA through the process of fluorescence resonance energy transfer (FRET) [29]. The phenomenon in question involves the transfer of excited-state energy from the donor FAM-ssDNA molecule to the acceptor NH_2_-PEG-rGO molecule. This transfer primarily occurs through a combination of surface adsorption and electron transfer, resulting in the quenching of the FAM-ssDNA sequence, also known as the ‘turn-off’ effect [30,31,32].

Furthermore, the versatile nature of NH_2_-PEG-rGO extends beyond its role as a support material for ssDNA immobilization, encompassing the facile adsorption of ssDNA on the rGO lattice, which involves both covalent and non-covalent interactions, with a particular emphasis on non-covalent pi-stacking interactions as the primary pathway. Conversely, double-stranded (ds) DNA exhibits a weaker binding affinity to the rGO surface compared to ssDNA, primarily because of the electrostatic repulsion from the phosphate backbone [33,34,35,36].

Building upon these findings, the current study aims to advance the investigation of ssDNA immobilization by substituting GO with NH_2_-PEG-rGO as the surface modification agent for NC membranes. The NC membrane modification with NH_2_-PEG-rGO was confirmed through Scanning Electron Microscopy (SEM), wettability assays, and Fourier Transform Infrared Spectroscopy (FTIR).

The NC/NH_2_-PEG-rGO hybrid membrane obtained underwent further testing for the adsorption of FAM-ssDNA from three distinct complex solutions. These tests were conducted using αMEM as the base medium with an incubation time of 60 min. Since ionic concentration plays a substantial role in establishing the attachment of ssDNA, these solutions included one with MgCl_2_, another with MnCl_2_, and a third one containing a combination of MgCl_2_ and MnCl_2_. Following the binding process, the membrane was washed with distilled water and then incubated in a desorption solution. This step facilitated the separation of FAM-ssDNA from the membrane and improved the affinity for extracting ssDNA.

## 2. Results and Discussion

### 2.1. Analysis of NC/NH_2_-PEG-rGO Hybrid Membrane

#### 2.1.1. FTIR Analysis

**Figure 1:**
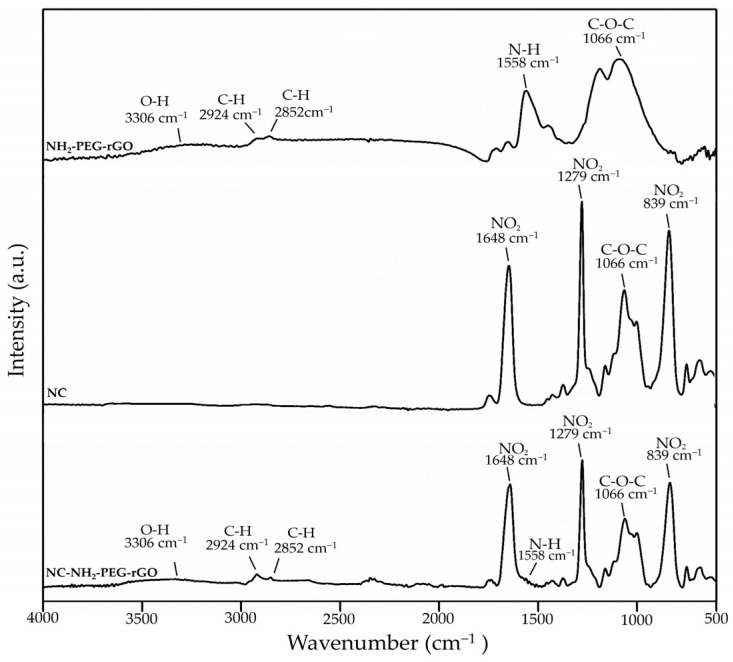
FTIR spectrum of NH_2_-PEG-rGO, NC, and NC/NH_2_-PEG-rGO hybrid membranes.

Through the molecular vibrations associated with each band, infrared spectroscopy imparts insight into the structures of the NC membrane modified with NH_2_-PEG-rGO investigated in the present study. The FTIR spectra for NH_2_-PEG-rGO, NC, and NC/NH_2_-PEG-rGO hybrid membranes are depicted in Figure 1.

Usually, the infrared spectrum of NH_2_-PEG-rGO is characterized by absorption peaks that are commonly found in the 1050–1085 cm^−1^, 1650–1540 cm^−1^, and 2800–3100 cm^−1^ sections [37,38]. These spectral bands are attributed to different vibrational modes, corresponding to C-O-C (epoxy groups), N-H (amine), and C-H (ethylene) groups. Furthermore, the spectral band at approximately 3300 cm^−1^ indicates the stretching vibrations of O–H (hydroxyl), in their free and unbound states [38,39,40,41]. In our particular situation, the mentioned absorption bands are displayed at 1066, 1558, 2852, and 2924 cm^−1^, respectively, as well as within the spectrum spanning from 3100 to 3400 cm^−1^. The FTIR spectrum of NC exhibits three main absorption peaks situated at 1648, 1279, and 839 cm^−1^, conceding to both symmetric and asymmetric stretching of the NO_2_ group [42,43,44].

The FTIR analysis of the NC/NH_2_-PEG-rGO hybrid membrane displays the characteristic absorption bands associated with both NH_2_-PEG-rGO and NC, confirming the successful fabrication of the NC/NH_2_-PEG-rGO hybrid membrane. The attendance of NH_2_-PEG-rGO is discernible through the faint absorption bands displayed at approximately 3300 cm^−1^, which are linked to the O-H groups within the rGO structure, as well as the C-H bending and stretching vibrations in the range of 2800–3100 cm^−1^, corresponding to the ethylene groups within the PEG structure [45,46]. Furthermore, the intensity of the distinctive absorption band corresponding to amino groups in the range of 1540–1600 cm^−1^ (N-H) appears reduced, likely due to the presence of NO_2_ functional groups in their proximity [37,47].

#### 2.1.2. Morphological Characterization

Before and after the adsorption procedure, morphological assessments were carried out on both NC and NC/NH_2_-PEG-rGO membranes.

**Figure 2:**
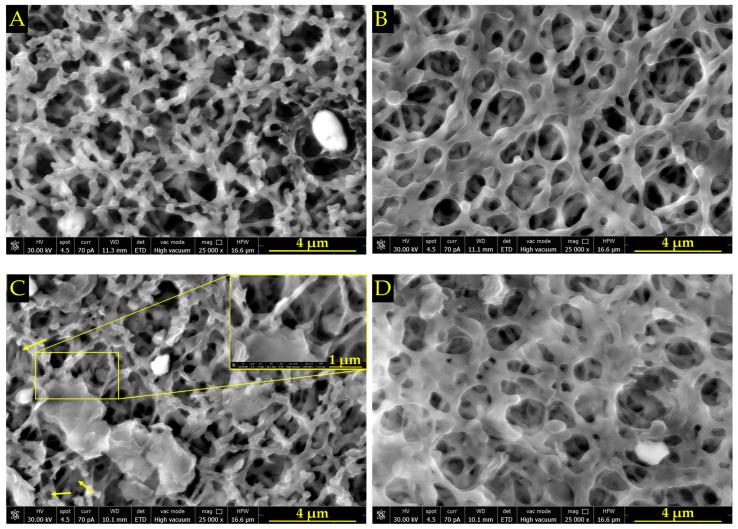
SEM micrographs that show the morphology of the NC membrane (**A**) before and (**B**) after incubation in the utilized ionic complex media, i.e., αMEM, SDS, BSA, FAM-ssDNA, and various ionic particles (MnCl_2_, MgCl_2_, and MnCl_2_/MgCl_2_). Additionally, the NC/NH_2_-PEG-rGO membrane is shown (**C**) before and (**D**) after incubation in the aforementioned media. All micrographs are presented with a 4 μm scale bar. The yellow arrows in subfigure (**C**) indicate single layers of NH_2_-PEG-rGO, and the inset displayed at a 1 μm scale bar reveals the agglomeration of NH_2_-PEG-rGO.

Prior to the experiment, the SEM micrograph from Figure 2A shows the NC membrane, which possessed a sponge-like structure with high porosity and interconnected open pores, characterized by surface irregularities. The observed homogeneity is consistent with the inherent porous nature of nitrocellulose, providing a baseline representation of the pristine membrane. Figure 2B captures the morphological changes in the NC membrane following the experimental incubation in the used media, i.e., αMEM, sodium dodecyl sulfate (SDS), bovine serum albumin (BSA), FAM-ssDNA, and various ionic particles (MnCl_2_, MgCl_2_, and MnCl_2_/MgCl_2_). The observed changes in morphology are twofold; on the one hand, the flattening of the NC membrane surface indicates moderate stability in aqueous media, leading to a more uniform surface morphology suggesting the efficient immobilization of FAM–ssDNA, and, on the other hand, a change in the pores’ shape and dimensions can be easily spotted, showing less uniform and larger pore diameters.

In Figure 2C, the morphology of the NC membrane can be observed under a very thin layer of NH_2_-PEG-rGO, showing features in some areas that indicate the natural flexibility of single layers of graphene, as indicated by the yellow arrows, but also occasional agglomeration of NH_2_-PEG-rGO (inset, Figure 2C). Following the experiment (Figure 2D), a flattening of the membrane morphology is observed, which occurred because of the FAM-ssDNA sticking to the membrane, making it appear denser and less transparent compared to the NC/NH_2_-PEG-RGO membrane and also to the pure NC membrane before the experiment. Furthermore, after the experiment, the surfaces of pure NC and NC/NH_2_-PEG-rGO appear distinct, with collapsed pores observed in pure NC and a clogged appearance evident in places on the NC/NH_2_-PEG-rGO hybrid membrane, likely indicating a greater affinity for the modified membrane to immobilize ssDNA compared to pure NC.

#### 2.1.3. Wettability Characteristics

**Figure 3:**
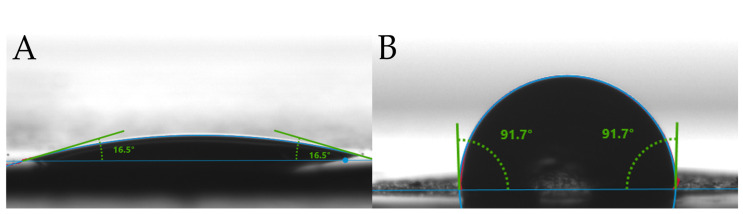
Water contact angles of (**A**) NC and (**B**) NC/NH_2_-PEG-rGO hybrid membrane.

The wettability characteristics of the NC and NC/NH_2_-PEG-rGO hybrid membranes are investigated using water contact angle analysis and are depicted in Figure 3. The measurements indicate distinct surface characteristics for the NC and the NC/NH_2_-PEG-rGO hybrid membrane. The NC membrane’s surface is hydrophilic, with a water contact angle of 16.5° (Figure 3A), indicating significant spreading of water droplets. This behavior suggests favorable wetting characteristics, highlighting the membrane’s hydrophilic nature [48,49].

In contrast, the NC/NH_2_-PEG-rGO hybrid membrane (Figure 3B) exhibits hydrophobic features, as shown by the water contact angles that exceed 90°. NH_2_-PEG-rGO modifies the surface of the NC membrane, resulting in reduced water attraction, with droplets resisting spreading, leading to a shift towards a more hydrophobic nature [50].

The characteristics exhibited by the surface of the NC/NH_2_-PEG-rGO hybrid membrane have a beneficial influence on how biomolecules engage with and respond to the membrane. The hydrophobic nature of NC/NH_2_-PEG-rGO facilitates hydrophobic interactions with biomolecules, such as lipid tails or aromatic rings, enhancing the membrane’s affinity for specific molecules. These interactions extend to molecules like ssDNA, where their hydrophobic regions, such as nucleotide bases, can engage in favorable interactions with the NC/NH_2_-PEG-rGO, thus enhancing the membrane’s affinity for ssDNA. Moreover, the NH_2_-PEG component adds specific chemical functionality to the surface, with amino (-NH_2_) groups facilitating covalent or electrostatic bonding with complementary chemical groups or charges from the ssDNA structure [51].

### 2.2. Oligo DNA Adsorption, Detection, and Extraction Using NC/NH_2_-PEG-rGO Hybrid Membranes in Complex Media

**Figure 4:**
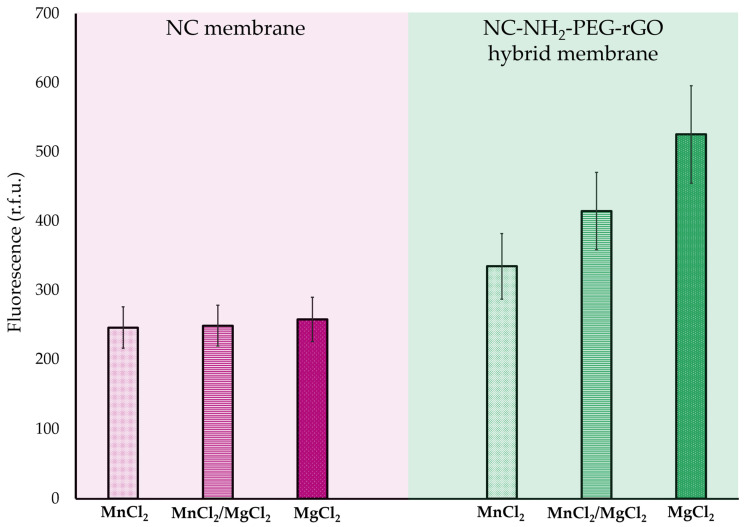
Measurement of FAM–ssDNA fluorescence intensity after 60 min of incubation on NC and NC/NH_2_-PEG-rGO membranes in complex solutions with various ionic particles (MnCl_2_, MnCl_2/_MgCl_2_, and MgCl_2_).

The efficiency of NC and NC/NH_2_-PEG-rGO hybrid membranes in detecting and extracting ssDNA was assessed by measuring fluorescence intensity after a 60-min incubation of the membranes in various ionic complex solutions containing MnCl_2_, MgCl_2_, or MnCl_2_/MgCl_2_. The resulting data are presented in Figure 4. Based on the results, the fluorescence intensity of the NC membrane in all ionic complex solutions does not exhibit any significant difference in immobilizing ssDNA, with the highest fluorescence recorded at approximately 250 r.f.u. for MgCl_2_ ionic solution.

In the context of the NC/NH_2_-PEG-rGO hybrid membrane, the measurements indicate a significantly greater affinity for immobilizing ssDNA in the complex solution containing MgCl_2_, resulting in a fluorescence intensity of approximately 525 r.f.u. In contrast, the complex solution containing the MnCl_2_ concentration demonstrates lower affinity, yielding a fluorescence intensity of around 330 r.f.u. While this affinity is lower than that observed with MgCl_2_, it still demonstrates the higher membrane capacity to interact with ssDNA in different conditions when compared with the NC membrane. The divalent cations Mn^2+^ and Mg^2+^ from the complex solution enhance the interactions between ssDNA and the NC/NH_2_-PEG-rGO membrane. This can occur because divalent cations can promote the condensation of ssDNA, reducing its exposure to the surrounding solvent and promoting interactions with hydrophobic surfaces [52,53]. The positively charged cations can interact with the negatively charged phosphate groups in the ssDNA backbone, neutralizing the negative charges and reducing electrostatic repulsion between ssDNA and the membrane surface, thereby facilitating its immobilization [54,55].

Furthermore, MnCl_2_ and MgCl_2_ can create specific binding sites on the membrane surface through their coordination chemistry. These binding sites act as anchor points for ssDNA, enhancing its immobilization in a site-specific manner. MnCl_2_ and MgCl_2_ interact with the DNA molecule, stabilizing its secondary and tertiary structures. This stabilization makes the ssDNA more rigid and structured, increasing its affinity for binding to the membrane. Additionally, divalent cations enhance ssDNA stability by strengthening hydrogen bonding, while amino groups from the hybrid membrane engage in specific hydrogen bonding interactions with ssDNA nitrogenous bases, thereby enhancing overall adsorption onto the membrane [56,57,58].

The divalent ions (Mg^2+^ and Mn^2+^) in the complex solutions demonstrate a higher affinity for immobilized ssDNA compared to the previous study [13], where the Na^+^ ions did not exceed values higher than 300 r.f.u. According to the Manning–Oosawa theory, approximately 88% of the DNA surface charge is neutralized by divalent counterions (Mg^2+^, Mn^2+^), while in the case of monovalent ions (Na^+^), this value is reduced to 76%, resulting in a lower charge density [59]. The lower charge density of monovalent ions means that they may not neutralize DNA’s negative charges as effectively as Mg^2+^ ions. The higher charge density of Mg^2+^ ions allows for more effective charge neutralization of DNA, reducing electrostatic repulsion and facilitating DNA–protein binding [59,60].

Various components, including serum proteins found within αMEM, particularly BSA and SDS, play a crucial role in reducing the non-specific binding of oligo ssDNA to diverse surfaces in the medium while simultaneously promoting its adherence to the membrane. BSA acts as a protective barrier, protecting the negatively charged oligo ssDNA molecules against repulsive electrostatic interactions with NC/NH_2_-PEG-rGO, while SDS deactivates nucleases and regulates non-specific adsorption on the surface of the NC/NH_2_-PEG-rGO hybrid membrane [61,62,63,64,65]. Moreover, αMEM incorporates glucose and other carbohydrates, leading to an increase in the solution’s osmotic pressure. This heightened osmotic pressure has the potential to induce a flow of water from the surrounding medium into the membrane, thereby enhancing the adsorption of oligo ssDNA onto the membrane’s surface [66,67].

The role of pH In modulating the interaction between ssDNA and the membrane is also important for understanding the binding dynamics. At Tris-HCl pH 8, the environment is slightly alkaline, resulting in partial deprotonation of both ssDNA and the NH_2_ groups on the membrane’s surface. This exposes negatively charged phosphate groups on the ssDNA backbone and positively charged amino groups on the membrane, promoting attractive electrostatic interactions between ssDNA and the membrane and facilitating ssDNA adsorption. On the other hand, at Tris-HCl pH 7, the environment is slightly acidic. This pH level can influence the charge state of both ssDNA and the membrane. The phosphate groups on the ssDNA backbone and the NH_2_ groups on the membrane surface are both partially protonated, resulting in a reduced net charge on both molecules. This reduction in charge weakens the electrostatic interactions between ssDNA and the membrane, making it easier for ssDNA to detach or desorb from the membrane surface [68,69,70,71].

However, considering the aforementioned phenomena, the immobilization of ssDNA to the surface of the modified membrane seems to be controlled by a series of factors that need to be specifically modulated: components in the ionic solution, ionic valence, and pH.

Table 1 illustrates the mass (measured in pg) of ssDNA desorbed from the NC and NC/NH_2_-PEG-rGO hybrid membranes after 60 min of incubation in the three complex media containing MnCl_2_, MnCl_2_/MgCl_2_, and MgCl_2_, respectively. In the case of the NC membrane, the results indicate that the desorbed mass does not significantly differ among the ionic complex used, ranging from 285 pg for MnCl_2_ to 300 pg for MgCl_2_. Conversely, for NC/NH_2_-PEG-rGO hybrid membranes, the highest mass detachment was observed in the case of MgCl_2_, approximately 610 pg, while the lowest was noted in the case of MnCl_2_, with approximately 390 pg. The slightly higher standard deviation obtained for the measurements is very likely attributable to the formation of seldom FAM-DNA aggregates within the solution. These findings suggest that, on average, a greater quantity of ssDNA is desorbed from NC/NH_2_-PEG-rGO hybrid membranes compared to the NC membrane.

The difference in the amount of ssDNA desorbed from the NC/NH_2_-PEG-rGO hybrid membranes in all the media used can be attributed to the fact that Mg^2+^ ions are smaller than Mn^2+^ ions, enabling them to neutralize the negative charges more effectively on the ssDNA molecule. Moreover, the smaller size of Mg^2+^ allows them to access and interact with binding sites on the membrane more effectively. Thus, smaller ions can fit into tighter spaces and reach sites that might be less accessible to larger ions, enhancing their binding efficiency [72,73].

Furthermore, both Mg^2+^ and Mn^2+^ ions are divalent cations, but Mg^2+^ ions have a higher charge density compared to Mn^2+^ ions. This higher charge density results in stronger electrostatic interactions with negatively charged functional groups on the membrane’s surface, such as oxygen atoms or other electronegative elements. This enhanced electrostatic attraction makes Mg^2+^ ions more likely to bind to the membrane. In addition to the charge density, Mg^2+^ ions are known to form stable coordination complexes with phosphate groups, which are abundant in ssDNA and RNA molecules. These complexes can enhance the binding of DNA to surfaces. While Mn^2+^ ions are capable of forming similar complexes, they may be less effective in binding ssDNA to the membrane due to their lower charge density [74,75,76].

Compared to our previous study where the NC-GO hybrid membrane emerged to extract approximately 335 pg of ssDNA with NaCl [13], in this case, the NC/NH_2_-PEG-rGO membrane implies a much higher extraction yield, with over 600 pg of ssDNA for the MgCl_2_ complex solution. This effect can be attributed to the presence of NH_2_ and polyethylene glycol (PEG) functional groups on the NC/NH_2_-PEG-rGO membrane that can enhance its ability to interact with and immobilize ssDNA molecules. These functional groups can provide additional binding sites and alter the surface charge of the membrane, potentially making it more favorable for ssDNA adsorption while leaving the graphene surface available for π–π interactions with ssDNA. The specific chemical composition and functional groups on the NC/NH_2_-PEG-rGO membrane may also favor interactions with Mg^2+^ ions compared to the interactions that occur between the NC-GO membrane and Na^+^ ions.

Although the NC/NH_2_-PEG-rGO membrane effectively extracts a higher quantity of ssDNA from these complex media containing various ionic particles, we recognize the necessity for further refinement. Undertaking additional research endeavors will facilitate the enhancement of both the efficiency and dependability of our technique, thereby expanding its versatility in the extraction of diverse biomolecule types from various sample sources. This advancement holds promise for the enrichment of molecular biology applications and other fields that depend on precise biomolecule extraction methods.

## 3. Materials and Methods

### 3.1. Reagents

Aminated Graphene Amino-PEG covalently linked, CAS No.: 7782-42-5, was procured from ACS-Materials (Pasadena, CA, USA). FAM-ssDNA was bought from Integrated DNA Technologies, Inc. (Coralville, IA, USA) and consists of the following base series: 5′-TTTCAACATCAGTCTGATAAGCTATCTCCC-3′, with labeling at the final primer using 6-carboxyfluorescein. The acquisition of the NC membranes characterized by a 47 mm diameter and an 8.0 µm pore size was purchased from Sartorius (Gottingen, Niedersachsen, Germany). αMEM, magnesium chloride (MgCl_2_), sodium dodecyl sulfate (SDS) with the chemical formula CH_3_(CH_2_)_11_OSO_3_Na, tris hydrochloride (Tris-HCl), and bovine serum albumin (BSA) were all sourced from Sigma-Aldrich, based in St. Louis, MO, USA. Manganese chloride (MnCl_2_) was procured from SILAL (Bucharest, Romania).

### 3.2. Preparation of Aminated Graphene Dispersion

The aminated graphene dispersion was obtained at a concentration of 1 mg/mL after 2 h of ultrasonication in an ice bath, using a VC×750 sonicator (Sonics & Materials, Inc., Newtown, CT, USA). This equipment operated at a 10-s pulse followed by a 5-s pause, and a frequency of 20 kHz.

### 3.3. NC/NH_2_-PEG-rGO Hybrid Membrane Fabrication

NC membranes were prepared with a 5 mm diameter and an approximate mass of 1.1 mg using a conventional paper hole punch. Non-covalent modification of the NC membrane was carried out using an approach akin to the dot blot technique. Initially, a diluted dispersion of NH_2_-PEG-rGO at a concentration of 400 µg/mL was prepared, and 5 µL of this dispersion was subsequently applied to the NC membrane through drop-casting. Prior to their utilization, the resulting hybrid NC/NH_2_-PEG-rGO membranes were left to air-dry overnight and cleaned with deionized water.

### 3.4. Adsorption, Extraction, and Detection of FAM-Labeled ssDNA in Ionic Solutions from NC/NH_2_-PEG-rGO Hybrid Membrane

Initially, three distinct ionic solutions were prepared for oligonucleotide immobilization using αMEM, each consisting of 100 mM MnCl_2_, 100 mM MgCl_2_, and a combination of MnCl_2_/MgCl_2_. These solutions were supplemented with 0.1 mg/mL BSA, 10 mM Tris-HCl at pH 8.0, 0.1% SDS, and 16 nM of FAM–ssDNA. The samples were spread at a volume of 100 µL per well into black Costar 96-well flat-bottomed plates.

**Figure 5:**
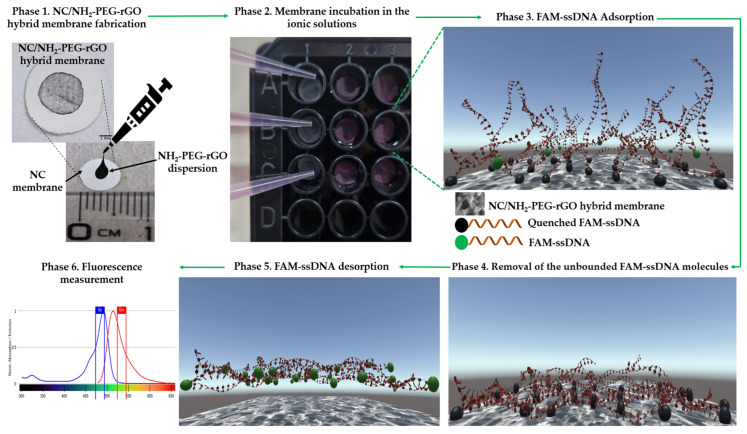
(Phase 1) Fabrication of the NC/NH_2_-PEG-rGO hybrid membrane by drop-casting a dispersion of NH_2_-PEG-rGO on the NC membrane; Phase 2 entails placing membranes into Costar 96-well flat-bottomed plates; Phase 3 involves immersing the membranes in solution containing FAM-ssDNA; Phase 4 involves removing the unbounded FAM-ssDNA molecules from the membrane surface; Phase 5 is dedicated to FAM-ssDNA desorption from NC/NH_2_-PEG-rGO hybrid membrane surface and fluorescence emission intensity measurements.

In the case of the adsorption approach, nine NC/NH_2_-PEG-rGO hybrid membranes were utilized (Phase 2 from Figure 5). Each membrane was incubated for 60 min in the prepared ionic solutions to facilitate the binding of FAM–ssDNA (Phase 3 according to Figure 5).

To eliminate any unbounded ssDNA, the membranes were washed with distilled water following incubation (phase 4 from Figure 5). Subsequently, the membranes were immersed in a 10 mM Tris-HCl solution at pH 7.0 for 45 min to initiate the FAM–ssDNA desorption process (phase 5 according to Figure 5). Following the removal of the NC/NH_2_-PEG-rGO hybrid membrane, the fluorescence of the desorbed solution was quantified using a microplate reader at a wavelength of 535 nm. The measurements were performed in triplicate for each membrane used, and the results were reported as average fluorescence values with the corresponding SD. Utilizing Equation (1), the resulting values were transformed into weight units and showcased in Table 1.

(1) $$M_{f}\;=\frac{F_{f}{\;\times \;M}_{i}}{F_{i}}$$

where *Mf* denotes the final mass of the released FAM–ssDNA in pg and *Ff* signifies the final fluorescence in r.f.u. following the desorption procedure. *Mi* is employed to represent the initial mass of the FAM–ssDNA in the complex solution (approximately 4420 pg), which was determined using the “DNA molecular weight and conversion” available through ThermoFisher (Waltham, MA, USA) [77]. Lastly, *Fi* denotes the solution fluorescence in r.f.u. after the introduction of the FAM–ssDNA sequence (around 3800 r.f.u.).

### 3.5. Spectrofluorimeter Assay

The fluorescence emission intensity was determined utilizing the TECAN Spark Fluorescence microplate reader (Tecan Trading AG., Männedorf, Switzerland) at a room temperature of 23 °C, with five readings recorded for each well at a wavelength of 535 nm.

### 3.6. Membrane Characterization

The FTIR studies used to investigate the interaction between NH_2_-PEG-rGO and NC were carried out using the ATR-FTIR spectrometer SHIMADZU 8900 equipment (Kyoto, Japan). Each FTIR spectrum was obtained within a range of 400–4000 cm^−1^, with 32 measurements taken for each sample and a resolution of 4 cm^−1^, and no additional sample preparation was required. To ensure the reproducibility of the results, all three samples, i.e., the NC membrane, the NH_2_-PEG-rGO powder, and the NC/NH_2_-PEG-rGO membrane, were studied in duplicate, and good agreement was found between spectra.

The NC membrane’s surface morphology was investigated using the FEI Quanta F250 scanning electron microscope. This examination was conducted both before and after the application of NH_2_-PEG-rGO particles, as well as before and after immersing the membranes in the complex solution. Before performing the SEM analysis, a thin stratum of gold-palladium was applied to increase the conductivity of the membranes.

Assessing the hydrophilicity of the membranes involved the application of the sessile drop method and the Krüss Scientific Drop Shape Analyzer-DSA100 (Hamburg, Germany). Investigating the impact of NH_2_-PEG-rGO on the hydrophilic characteristics of the NC membrane included conducting static water contact angle measurements at an ambient temperature. Capturing the configuration of the deionized water droplet on the specimen surface utilized a CF03 digital camera over a 5-s interval following the deposition of a 2 μL droplet. The determination of water contact angle values involved using DSA3 software (version 2-11) and constituted the mean of three measurements for each specimen. The interpretation of outcomes employed the Young–Laplace equation [78].

## 4. Conclusions

In the present study, we conducted an investigation using an NC/NH_2_-PEG-rGO membrane for oligonucleotide extraction. The structural and morphological features obtained by FTIR and SEM investigations demonstrated the successful fabrication of the hybrid membrane. SEM microscopy unveiled a sponge-like structure under a very thin layer of graphene with seldom agglomerations, showcasing the effective dispersion and uniform coating of NH_2_-PEG-rGO on the NC membrane. The wettability characteristics revealed that the NC/NH_2_-PEG-rGO hybrid membrane displayed a significantly higher hydrophobic water contact angle at 91.7° compared to the NC control membrane, which exhibited a contact angle of 16.5°. The higher hydrophobic character of the membrane is believed to have a beneficial effect on nucleic acid adsorption.

The fluorescence emission intensity results demonstrate that the NC/NH_2_-PEG-rGO hybrid membrane consistently adsorbed and desorbed oligonucleotides across three complex media with different ionic compositions (MnCl_2_, MnCl_2_/MgCl_2_, and MgCl_2_). Notably, the medium containing MgCl_2_ exhibited the most favorable outcome.

In the case of the control, the NC membrane exhibited a reduced capacity to adsorb ssDNA, resulting in the lowest fluorescent intensity of approximately 250 r.f.u. in the media with MnCl_2_, which corresponds to a quantity of 286 pg. The data obtained suggest that modifying the NC membrane with NH_2_-PEG-rGO substantially improves its interaction with nucleic acids, resulting in a significantly higher binding affinity.

After incubating the NC/NH_2_-PEG-rGO hybrid membrane in complex media with MgCl_2_, the highest fluorescent intensity displayed a signal above 520 r.f.u., corresponding to a quantity of 611 pg of ssDNA. However, when incubated in complex media with MnCl_2_, the fluorescent signal decreased to 330 r.f.u., corresponding to 290 pg. Moreover, in any type of media used, the NC/NH_2_-PEG-rGO can immobilize a higher number of oligonucleotides than the NC membrane.

Hence, our research outcomes strongly indicate that when the NC membrane is modified with NH_2_-PEG-rGO, it enables a substantially increased immobilization capacity for oligo-DNA on the membrane surface. This significant enhancement in oligo-DNA immobilization underscores the potential of NH_2_-PEG-rGO as a superior choice for membrane modification, with profound implications for applications demanding efficient oligo-DNA immobilization and extraction.

## Figures and Tables

**Table 1:** Data of ssDNA mass (pg) and standard deviation (s.d.) after 60 min of incubation on the NC and NC/NH_2_-PEG-rGO hybrid membranes in complex media with various ionic particles (MnCl_2_, MnCl_2_/MgCl_2_, and MgCl_2_).

Ionic Particles in Complex Media	Desorption of ssDNA from the NC Membrane Measured in pg, with Standard Deviation (s.d.)		Desorption of ssDNA from the NC/NH_2_-PEG-rGO MEMBRANE Measured in pg, with Standard Deviation (s.d.)	
Time	60 min			
U/M	pg	s.d.	pg	s.d.
MnCl_2_	286.41	±34.75	389.32	±54.98
MnCl_2_/MgCl_2_	289.67	±34.4	482.35	±65.23
MgCl_2_	300.25	±37.1	611.12	±82.03

U/M stands for Unit of Measurement.

## Data Availability

Data are contained within the article.
